# The incidence of microvascular obstruction with acute myocardial infarction and the relationship between the occurrence of microvascular obstruction and infarct size, angiographic findings and clinical background: gadolinium-contrasted MRI study

**DOI:** 10.1186/1532-429X-11-S1-P234

**Published:** 2009-01-28

**Authors:** Masashi Kawade, Kunihiko Teraoka, Shintaro Kiuchi, Yoshinori Suzuki, Kenji Takazawa, Akira Yamashina

**Affiliations:** Tokyo Med. University, Tokyo, Japan

**Keywords:** Acute Myocardial Infarction, Infarct Size, Acute Stage, Regional Wall Motion, Angiographic Finding

## Introduction

It has been shown that the presence of microvascular obstruction in humans after myocardial infarction is associated with poor prognosis and worse left ventricle remodeling. Even now it is not unclear to avoid the occurrence of MO as the reperfusion injury with reperfusion therapy and to treat it.

## Purpose

To clarify the background of the occurrence of MO, the incidence of MO with AMI pts in the acute phase was evaluated, and the relationship between the infarct size and coronary angiographic findings, and clinical backgrounds, regional wall motion were investigated.

## Methods

Cardiac MR was performed in 90 patients (male/female; 72/18, mean age was 61.3 +/- 11.8 years old) within 7 days from the onset of AMI(6 +/- 2.4 days). Especially 25 cases were followed and examined 2^nd^ CMR at 6 months later. All pts were treated with PCI successfully and their final TIMI grade were TIMI III. The MO quantification was determined by perfusion MRI and LGE techniques.

## Results

**Figure 1:**
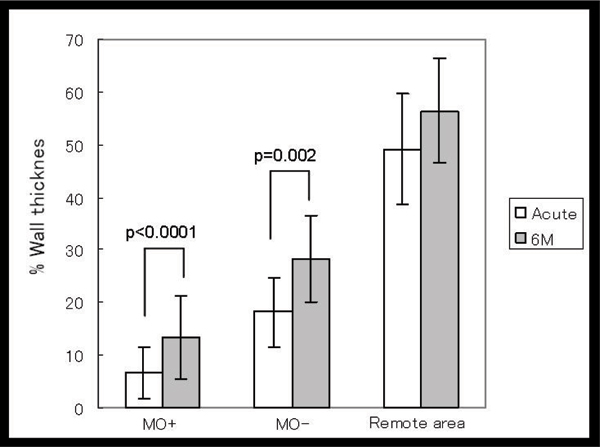
Figure 1

**Figure 2:**
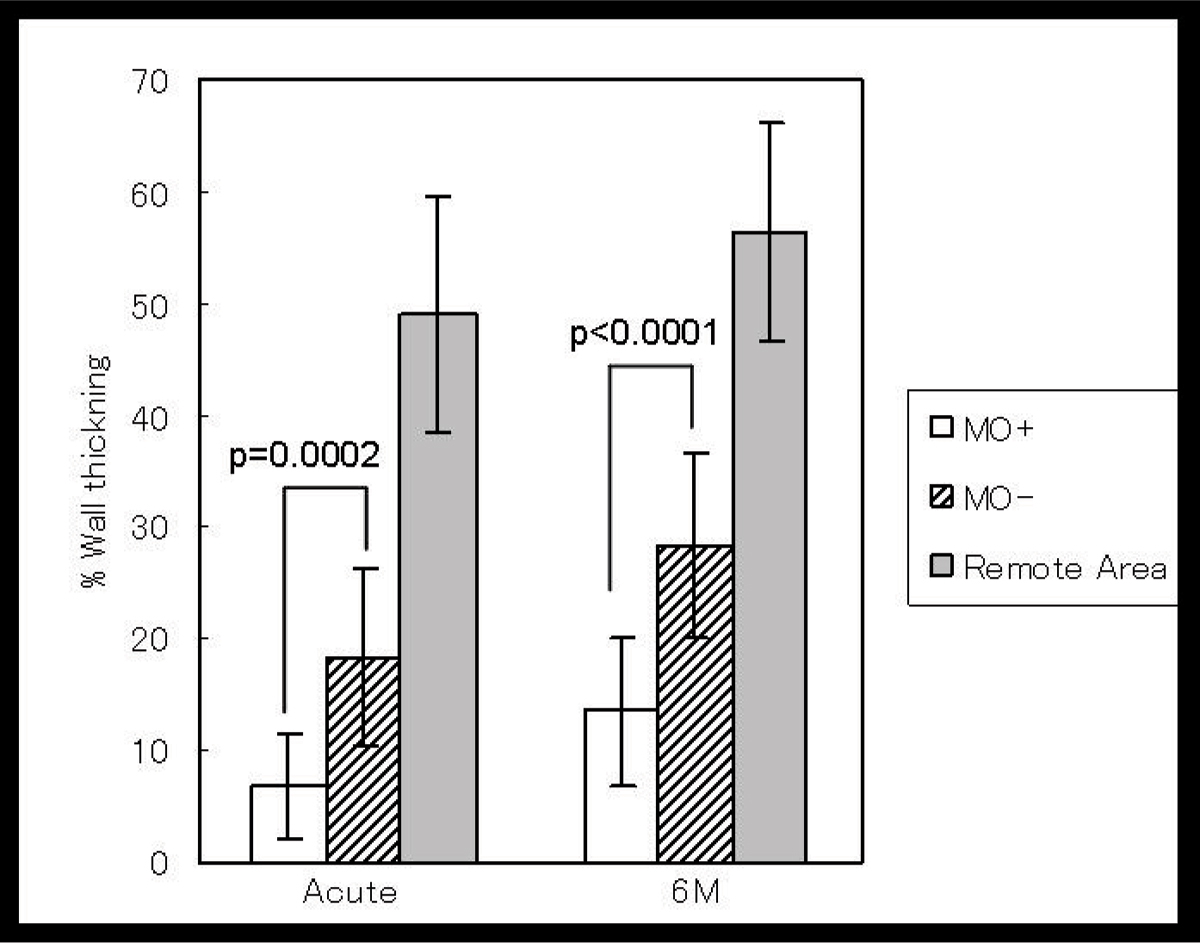
Figure 2

1) MO was recognized in 53 patients (58.9%) with the both techniques. 2) Infarct area evaluated by LGE was more extended circumferentially (P < 0.0001) and also more extended from endocardium to epicardium in MO positive patients than negatives significantly, (P < 0.0001). 3) Peak CK level was also higher in MO positive patients than negatives significantly (P < 0.0001). 4) The time from onset to revascularization, there was a difference in neither. But blush grade and collateral index were lower in MO positive patients than negatives significantly (p = 0.00058, p = 0.046, respectively). 5) There was higher incidence of heart failure in hospitalization of MO positive patients than negatives but not significantly (Fisher' test. p = 0.078) 6) The segments showed MO impaired segmental wall thickening (SWT) at acute stage compared to the segments without MO. The SWT remained impaired at the segments with MO, compared to the segments without MO at 6 months later with 25 cases follow up (Figures 1, 2).

## Conclusion

In acute stage of AMI, MO was recognized in the patients having large infarction, worse angiographic findings and also the high incidence of heart failure.

